# Examining the genetic relationship between Alzheimer’s disease, schizophrenia and their shared risk factors using genomic structural equation modelling

**DOI:** 10.1093/braincomms/fcaf112

**Published:** 2025-04-10

**Authors:** Inês Almeida e Sousa, Andrew D Grotzinger, Jeremy M Lawrence, Sophie Breunig, Charles R Marshall, Ania Korszun, Isabelle F Foote

**Affiliations:** Unit for Psychological Medicine, Wolfson Institute of Population Health, Queen Mary University of London, London EC1M 6BQ, UK; Centre for Preventive Neurology, Wolfson Institute of Population Health, Queen Mary University of London, London EC1M 6BQ, UK; Institute for Behavioral Genetics, University of Colorado Boulder, Boulder, CO 80303, USA; Department of Psychology and Neuroscience, University of Colorado Boulder, Boulder, CO 80309, USA; Institute for Behavioral Genetics, University of Colorado Boulder, Boulder, CO 80303, USA; Department of Psychology and Neuroscience, University of Colorado Boulder, Boulder, CO 80309, USA; Institute for Behavioral Genetics, University of Colorado Boulder, Boulder, CO 80303, USA; Department of Psychology and Neuroscience, University of Colorado Boulder, Boulder, CO 80309, USA; Centre for Preventive Neurology, Wolfson Institute of Population Health, Queen Mary University of London, London EC1M 6BQ, UK; Unit for Psychological Medicine, Wolfson Institute of Population Health, Queen Mary University of London, London EC1M 6BQ, UK; Unit for Psychological Medicine, Wolfson Institute of Population Health, Queen Mary University of London, London EC1M 6BQ, UK; Centre for Preventive Neurology, Wolfson Institute of Population Health, Queen Mary University of London, London EC1M 6BQ, UK; Institute for Behavioral Genetics, University of Colorado Boulder, Boulder, CO 80303, USA

**Keywords:** Alzheimer’s disease, schizophrenia, dementia, psychosis, genomic structural equation modelling

## Abstract

Epidemiological studies have demonstrated an association between dementia and schizophrenia. There is a significant symptom overlap between the two disorders—psychosis is seen in 50% of patients with Alzheimer’s disease and cognitive impairment is a key feature of schizophrenia. Whether these overlapping clinical presentations reflect shared aetiology is unclear. Therefore, we aimed to model the genetic correlation between Alzheimer’s disease, schizophrenia and their shared risk factors using genomic structural equation modelling to identify potentially overlapping biological pathways between these traits. We measured genetic correlation between Alzheimer’s disease, schizophrenia and 13 shared risk factors, including body fat percentage, less education, alcohol intake, insomnia, loneliness, less social/leisure activity, major depression, mean arterial pressure, smoking, socioeconomic deprivation, low-density lipoprotein cholesterol, eye problems and type 2 diabetes mellitus. Schizophrenia and Alzheimer’s disease were not significantly genetically correlated but were both significantly associated with loneliness. Colocalization suggested that the association between loneliness and Alzheimer’s disease was predominantly driven by a shared causal variant on Chromosome 11. Factor analysis of shared risk factors produced four latent factors representing clusters of shared genetics between socioeconomic traits, psychiatric traits, cardiometabolic traits and smoking-related traits. Both Alzheimer’s disease and schizophrenia were significantly associated with the socioeconomic latent factor. Although there is little direct genetic overlap between schizophrenia and Alzheimer’s disease, loneliness may play an important role in the association between these two disorders. In addition, the shared genetics between socioeconomic traits may affect susceptibility to both Alzheimer’s disease and schizophrenia to a greater extent than trait-specific pathways.

## Teaser

Almeida e Sousa *et al*. report that although there is no direct genetic correlation between Alzheimer’s disease and schizophrenia, both disorders are genetically correlated with loneliness and the genetic overlap that underpins socioeconomic-related risk factors. This study helps to refine our knowledge of the pleiotropic pathways between schizophrenia and dementia.

## Graphical Abstract

**Graphical Abstract:**
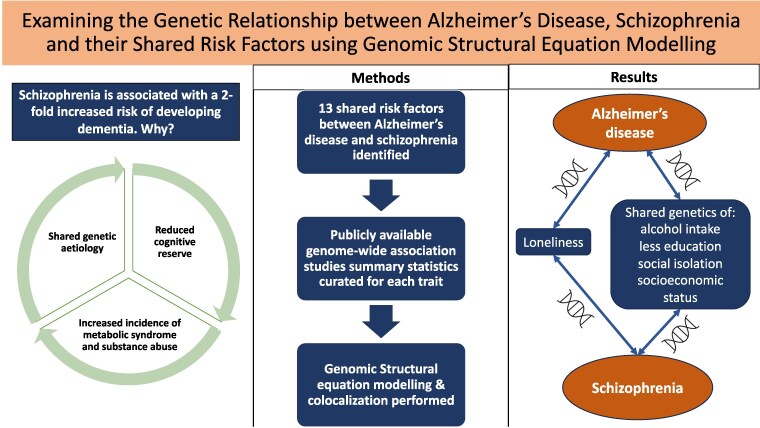

## Introduction

Dementia and schizophrenia are clinically distinct chronic brain disorders. Despite the differences in clinical presentation, age of onset and molecular pathways involved, converging evidence supports an association between schizophrenia and dementia. Individuals with schizophrenia have a 2-fold increased risk of developing dementia compared with the general population^1^ and are also more likely to develop dementia earlier.^2,3^ A few possible explanations for this association have been proposed. First, there is symptom overlap between the clinical presentations of dementia and schizophrenia (including psychosis and cognitive impairment), which may reflect a shared aetiology between them.^1,4^ Second, it has been hypothesized that patients with schizophrenia have a reduced cognitive reserve, which, when coupled with the accelerated brain ageing seen in these patients, may put them at a higher risk of developing dementia.^2,5^ Third, schizophrenia is associated with an increased risk of cardiovascular disease, metabolic syndrome, social isolation, smoking and substance abuse, which are hypothesized dementia risk factors.^6-9^ This suggests that schizophrenia may associate with dementia because it independently increases the prevalence of modifiable risk factors that are in turn associated with a greater risk of cognitive decline later in life.

Both Alzheimer’s disease, the commonest cause of dementia, and schizophrenia are highly heritable traits with an estimated heritability of 71% (for late-onset Alzheimer’s disease) and 80%, respectively.^10,11^ Furthermore, genome-wide association studies (GWASs) have identified numerous genomic risk loci for both disorders, including 75 loci for Alzheimer’s disease and 287 loci for schizophrenia in recent studies.^12,13^ Therefore, in the present study, we used genomic structural equation modelling (Genomic SEM), which uses GWAS data, to compare how schizophrenia and Alzheimer’s disease relate to each other and their shared risk factors on a genetic level. We used Alzheimer’s disease as our phenotype for dementia because this is currently the only clinical subtype of dementia where the available GWAS summary data provide enough power to be reliably included in Genomic SEM analyses. The Brainstorm consortium demonstrated that neurodegenerative and psychiatric traits are genetically distinct, but that both Alzheimer’s disease and schizophrenia are genetically correlated with lower levels of education.^14^ Therefore, we hypothesized that whilst the direct genetic correlation between Alzheimer’s disease and schizophrenia would be small,^14,15^ there would be a high genetic correlation between the risk factors and including this in our multivariate modelling framework may help to uncover pleiotropic pathways between the two disorders.^16^

## Materials and methods

### Phenotype selection and data curation

We identified publicly available GWAS summary statistics for all included phenotypes. Selected GWAS datasets had a sample size of ≥10 000, included samples of unrelated individuals of European ancestry, were non-sex stratified and did not adjust for heritable covariates.^15,17^

#### Schizophrenia and Alzheimer’s disease

We used the largest published GWAS for schizophrenia from the Psychiatric Genomics Consortium, consisting of 55 193 cases and 74 132 controls.^13^ For our dementia phenotype, we used GWAS summary statistics for Alzheimer’s disease, since this is the most common cause of dementia globally and has the largest publicly available GWAS samples of any dementia subtype to date. We used data from the Lambert *et al*.^18^ GWAS that included 17 008 cases and 37 154 controls. We note that we used data from this Alzheimer’s disease GWAS for our main analysis because it had comparable SNP-based heritability Z-statistic estimates compared with the later Kunkle *et al*.^19^ GWAS (4.38 versus 4.78). However, it displayed larger mean absolute genetic correlations with the other included phenotypes [mean $\;r_{g}\;$ = 0.04 (Lambert) versus 0.02 (Kunkle)], meaning that it was more appropriate to use for Genomic SEM since more of the Alzheimer’s disease SNP-based heritability overlapped with the SNP-based heritability of the risk factors. This discrepancy is likely because the measured SNPs were different between the two GWASs, which could lead to divergent mean genetic correlations with external risk factor traits because the overall genetic correlation estimate will be the average of different patterns of local genetic correlation across the genome. Nevertheless, we also replicated the main analyses using the larger GWAS by Kunkle *et al*. (21 982 cases and 41 944 controls) to compare the findings across Alzheimer’s disease samples (Supplementary Results and Supplementary Tables 12–13). However, we did not use the more recent Alzheimer’s disease GWAS by proxy (GWAX) samples due to the inclusion of proxy Alzheimer’s disease cases, which represent a less clinically relevant and more heterogeneous Alzheimer’s disease phenotype that may result in a more diffuse GWAS signal.^12,20-22^

#### Risk factors

We identified GWAS summary statistics for potentially modifiable risk factors that are shared between schizophrenia and dementia. We used the Lancet Commission on Dementia Prevention, Intervention and Care 2024^6^ report to inform our inclusion of dementia risk factors and reviewed the literature to assess whether they were also associated with schizophrenia (Supplementary Materials).

This resulted in the inclusion of 13 shared risk factor traits reflecting body fat percentage, less education, alcohol intake, insomnia, loneliness/isolation, less social/leisure activity, major depressive disorder, mean arterial pressure, smoking, socioeconomic deprivation, low-density lipoprotein (LDL) cholesterol, diagnosis of an eye disorder/problem and type 2 diabetes mellitus^23-27^ (see Supplementary Table 1 and Supplementary Materials for detailed sample descriptions). We note that whilst insomnia and socioeconomic deprivation were not included within the central 14 risk factors of the Lancet Commission, sleep disturbance and increased deprivation were highlighted to be additional putative risk factors for dementia, so they were included in the current study since they have also been associated with schizophrenia risk.^28,29^

#### Quality control

We formatted each of the univariate GWAS phenotypes using the ‘munge’ function of the *GenomicSEM* R package.^30^ This step aligned the effect and non-effect alleles across all the measured phenotypes using the HapMap3 SNPs as our reference panel. We also filtered each GWAS phenotype to remove poorly imputed SNPs (imputation score ≥0.9) and rare variants (minor allele frequency ≥0.01).^30^

### Statistical analysis

#### Multivariable linkage disequilibrium score regression

We performed a multivariable version of linkage disequilibrium (LD) score regression using the *GenomicSEM* R package^30^ to estimate the SNP-based heritability ($h_{\mathrm{SNP}}^{2})$ of each included trait and the genetic correlation ($r_{g}$) between each pair of traits.^30^ This method estimates the genetic covariance matrix and the associated sampling covariance matrix between all included traits. We used publicly available pre-computed LD scores and weights based on European ancestry data from 1000 Genomes project Phase 3. The SNP-based heritability of binary traits was estimated on the liability scale using population prevalence estimates from recent epidemiological studies (Supplementary Table 1 and Supplementary Methods). In cases where the trait was binary and consisted of data from a meta-analysis of multiple cohorts, we calculated the sum of effective sample size and used a sample prevalence estimate of 0.5 to correct for ascertainment bias, which has been shown to produce more accurate heritability estimates.^31^ The heritability of continuous traits was estimated on the observed scale. We used this step to confirm the heritability *Z*-statistics were >4 for each trait to ensure that the traits were adequately powered to produce interpretable estimates of genetic overlap with other traits.^17^

#### Genomic SEM

We used the genetic covariance and corresponding sampling covariance matrices from the multivariable LD score regression to test a series of model comparisons using Genomic SEM. We initially standardized these matrices into genetic correlation and sampling correlation matrices, so that the results reflected the more interpretable proportion of genetic overlap between the modelled traits.

We estimated fully saturated models for the 13 risk factors to measure the amount of genetic overlap between Alzheimer’s disease, schizophrenia and each risk factor, using a false discovery rate (FDR)-corrected *P*-value <0.05. Next, we specified a constrained model for each risk factor where the genetic overlap for Alzheimer’s disease and schizophrenia with the risk factor was constrained to be equal. Here, the *P*-value for the constrained model $\chi^{2}$ statistic signifies the decrement in model fit compared with the fully saturated model allowing us to assess whether Alzheimer’s disease and schizophrenia have significantly different patterns of genetic overlap with each risk factor. We again used an FDR-corrected *P*-value threshold of <0.05 to account for multiple testing.

#### Colocalization

In the case of loneliness, we found significant genetic overlap with both Alzheimer’s disease and schizophrenia. To explore these associations further, we performed colocalization using the Coloc R Package to test whether any of the genomic risk loci for loneliness shared the same causal variant with schizophrenia or Alzheimer’s disease.^32^ Genomic risk loci were defined using the default parameters in FUMA v1.3.6,^33^ which identified 16 genomic risk loci for loneliness (see Supplementary Table 2). All variants within a 1-Mb window of the lead variant for each of the loneliness genomic risk loci were included in the analysis. In cases where the lead variant was not present in the schizophrenia or Alzheimer’s disease GWAS, an LD proxy variant was used where possible. We used the default prior probabilities for all analyses, whereby the prior probability of an association with either loneliness (p1) or the disease outcome (schizophrenia or Alzheimer’s disease) (p2) was set at 1 × 10^−4^ and the prior probability of an association with both traits (p12) was set at 1 × 10^−5^.^32^ Regions that had a posterior probability ≥0.75 were considered to show evidence for colocalization (i.e. a shared causal SNP in the case of PPH_4_) or pleiotropy driven by distinct causal variants in the same genomic region (PPH_3_).

#### Follow-up genomic factor analysis of risk factors

Previous studies have demonstrated that dementia modifiable risk factors are highly genetically correlated.^16^ Therefore, we also conducted a genomic factor analysis to assess how the shared genetic component between risk factors relates to Alzheimer’s disease and schizophrenia. We used the genetic covariance matrix and associated sampling covariance matrix from the multivariable LD score regression to measure the pairwise genetic correlations ($r_{g}$) between the 13 risk factor traits using an FDR-corrected *P*-value threshold of <0.05.

Since we did not know the latent genetic patterns underlying this set of 13 risk factors, we initially conducted an exploratory factor analysis (EFA) to use as the foundation for a follow-up confirmatory factor analysis (CFA). To avoid model overfitting, we conducted the EFA in the odd autosomes and the CFA in the even autosomes.^16^ We used multivariable LD score regression to calculate the corresponding genetic covariance matrices and the associated sampling covariance matrices for these chromosomal groupings. We used the Kaiser rule to define the number of factors to extract and then used the results from the EFA to specify our CFA model, including only standardized factor loadings ≥0.30. The CFA was performed using diagonally weighted least square estimation, which considers the varying precision of the genetic estimates when estimating the model.^30^

We then tested the overall genome-wide model fit of the CFA in all autosomes, and using the genome-wide correlation matrix and sampling correlation matrix, the same set of model comparisons for the single risk factors was performed, wherein the decrement in model fit was evaluated when constraining the genetic correlation to be equal between the latent factors and Alzheimer’s disease and schizophrenia. We used an FDR-corrected *P*-value threshold of <0.05 to correct for four tests.

## Results

**Figure 1:**
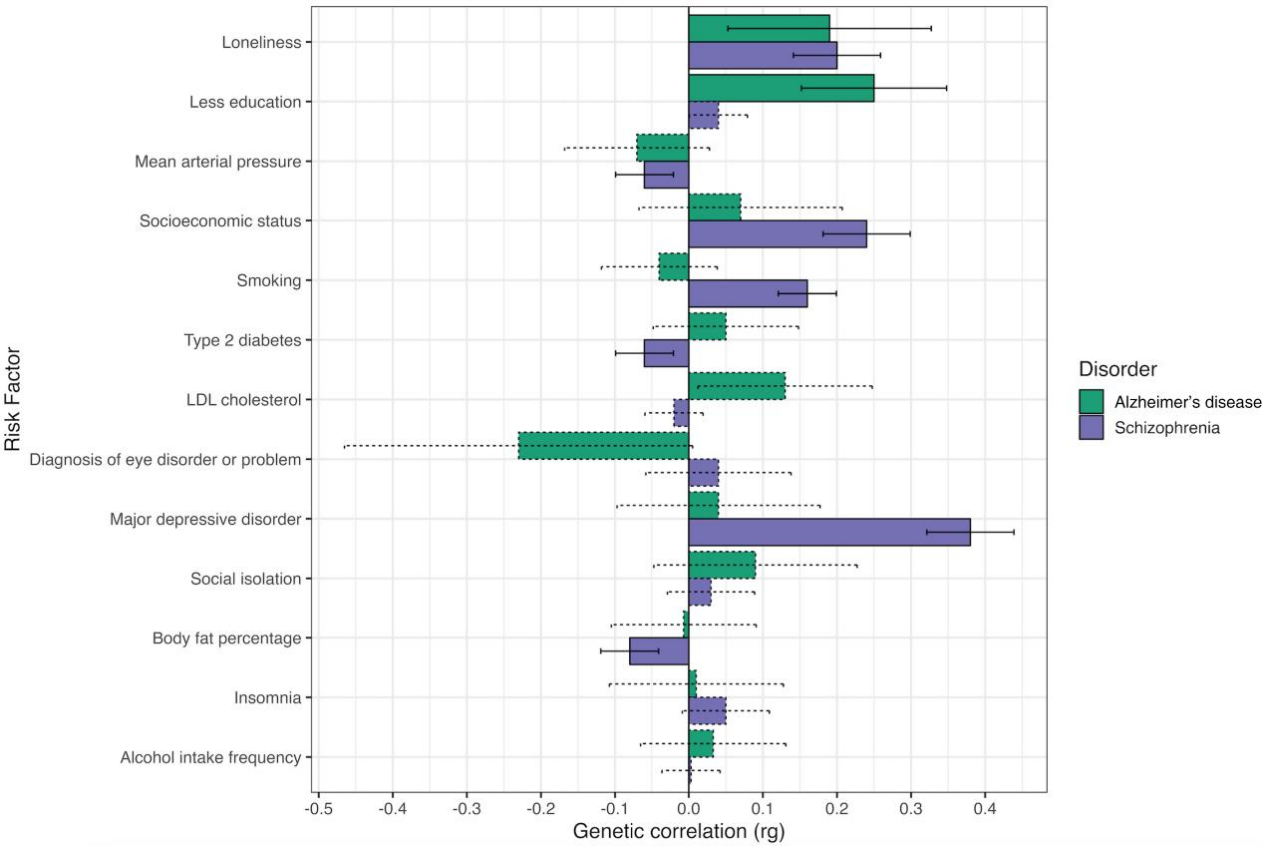
**A comparison of the genetic correlations between Alzheimer’s disease and schizophrenia and risk factors estimated using multivariable LD score regression.** Solid bars represent significant genetic correlations passing FDR-corrected *P*-value <0.05, whereas dashed lines represent non-significant genetic correlations. Error bars represent the 95% confidence intervals. These analyses used GWAS summary statistics data for Alzheimer’s disease *(n =* 54 162), schizophrenia *(n =* 129 325), loneliness (*n* = 413 936), less education (*n* = 416 316), mean arterial pressure (*n* = 417 001), socioeconomic status (*n* = 420 035), smoking (*n* = 632 802), type 2 diabetes (*n* = 936 700), LDL cholesterol (*n* = 398 402), diagnosis of eye disorder/problem (*n* = 135 363), social isolation (*n* = 419 219), major depressive disorder (*n* = 173 005), body fat percentage (*n* = 412 960), insomnia (*n* = 386 988) and alcohol intake frequency (*n* = 420 008).

Multivariable LD score regression found a non-significant genetic correlation between Alzheimer’s disease and schizophrenia ($r_{g}$ = 0.05, SE= 0.05, *P* = 2.95 × 10^−01^). All included phenotypes had $h_{\mathrm{SNP}}^{2}$  *Z*-statistics >4 and were therefore brought forward for subsequent analysis (Supplementary Table 3). Schizophrenia was significantly genetically correlated with increased loneliness ($r_{g}$ = 0.20; SE = 0.03, *P*_FDR_ = 2.95 × 10^−11^), lower mean arterial pressure ($r_{g}$ = −0.06, SE = 0.02, *P*_FDR_ = 9.23e-03), negatively correlated with type 2 diabetes mellitus ($r_{g}$ = −0.06, SE = 0.02, *P*_FDR_ = 9.23 × 10^−03^), lower socioeconomic status ($r_{g}$ = 0.24, SE = 0.03, *P*_FDR_ = 1.90 × 10^−17^), increased risk of major depressive disorder ($r_{g}$ = 0.38, SE = 0.03, *P*_FDR_ = 2.25 × 10^−34^), smoking ($r_{g}$ = 0.16, SE = 0.02, *P*_FDR_ = 5.07 × 10^−11^) and decreased body fat percentage ($r_{g}$ = −0.08, SE = 0.02, *P*_FDR_ = 4.32 × 10^−05^). By comparison, Alzheimer’s disease evinced a more limited pattern of genetic overlap, with significant genetic correlations identified with increased loneliness ($r_{g}$ = 0.19, SE = 0.07, *P*_FDR_ = 1.95 × 10^−02^) and lower educational attainment ($r_{g}$ = 0.25, SE = 0.05, *P*_FDR_ = 2.17 × 10^−06^). Most risk factors demonstrated genetic correlations in the same direction of effect for both Alzheimer’s disease and schizophrenia, except for type 2 diabetes mellitus, smoking, LDL cholesterol and diagnosis of eye disorder or problem (Fig. 1, Supplementary Table 4 and Supplementary Materials).

The results from our constrained models demonstrated significantly divergent genetic correlations between Alzheimer’s disease and schizophrenia for major depression (Alzheimer’s disease: $r_{g}$ = 0.04, SE = 0.07; schizophrenia: $r_{g}$ = 0.38, SE = 0.03; model $\chi^{2}\;$ difference *P*_FDR_ = 5.59 × 10^−05^), less education (Alzheimer’s disease: $r_{g}$ = 0.25, SE = 0.05; schizophrenia: $r_{g}$ = 0.04, SE = 0.02; model $\chi^{2}\;$ difference *P*_FDR_ = 7.73 × 10^−04^) and smoking (Alzheimer’s disease: $r_{g}$ = −0.04, SE = 0.04; schizophrenia: $r_{g}$ = 0.16, SE = 0.02; model $\chi^{2}\;$ difference *P*_FDR_ = 2.67 × 10^−04^). By contrast, loneliness was significantly genetically correlated with both disorders and the magnitude of the genetic correlation was not found to significantly differ between disorders (Alzheimer’s disease: $r_{g}$ = 0.19, SE = 0.07; schizophrenia: $r_{g}$= 0.20, SE = 0.03; model $\chi^{2}\;$ difference *P*_FDR_ = 9.58 × 10^−01^) (Supplementary Table 4).

**Figure 2:**
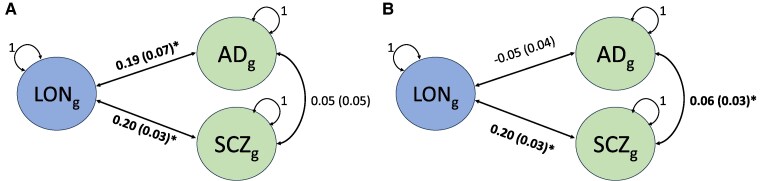
**Path diagrams of the fully saturated models for (A) the genome-wide genetic association between loneliness, schizophrenia and Alzheimer’s disease and (B) the genetic association between loneliness, schizophrenia and Alzheimer’s disease with the rs12364432 region removed.** These constrained models were estimated using Genomic SEM. The left-hand circle in each model depicts the genetic variance of loneliness (LON, *n* = 413 936), whereas the right-hand circles depict the genetic variance of Alzheimer’s disease (*n =* 54 162) and schizophrenia (*n =* 129 325). Standardized correlation estimates are provided with their standard errors in parentheses. Statistically significant genetic correlations (*P* < 0.05) are highlighted in bold with an asterisk.

To examine this relationship with loneliness further, we ran a colocalization analysis to determine whether there was any evidence of pleiotropy between loneliness and schizophrenia (Supplementary Table 5) or Alzheimer’s disease (Supplementary Table 6) that could be driving these associations. We found strong evidence for a shared causal variant (PPH_4_ = 0.90) between loneliness and schizophrenia at the rs159960 locus on Chromosome 1, but this region did not show evidence for pleiotropy with Alzheimer’s disease (PPH_1_ = 0.82). By contrast, we found strong evidence for a shared causal variant (PPH_4_ = 0.75) between loneliness and Alzheimer’s disease at the rs12364432 locus on Chromosome 11 [genomic location of locus (build 37): chr11:47372377–chr11:47946836], whereas the association with schizophrenia at this locus is likely to be driven by independent causal variants at this locus (PPH_3_ = 1.0). Recalculation of the constrained model in Genomic SEM with this region removed demonstrated that whilst the genetic association between schizophrenia and loneliness remained comparable to the genome-wide model ($r_{g}$ = 0.20, SE = 0.03, *P* = 6.68 × 10^−11^), the genetic association with Alzheimer’s disease was completely attenuated ($r_{g}$ = −0.05, SE = 0.04, *P* = 2.49 × 10^−1^) (Fig. 2).

**Figure 3:**
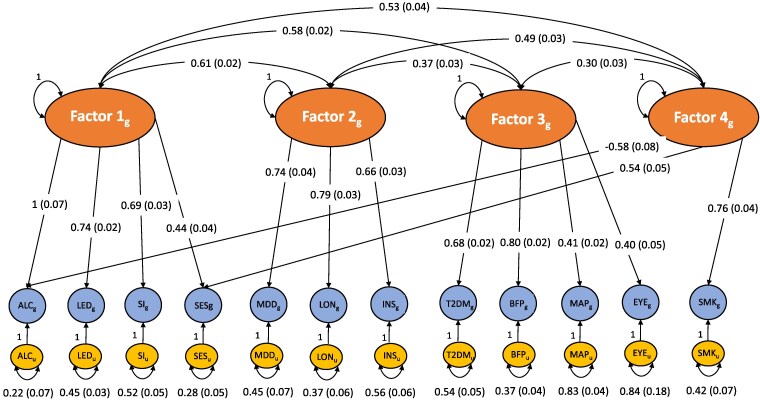
**Path diagram of the four-factor model estimated using data from all autosomes (i.e. Chromosomes 1–22) estimated by CFA.** The large ovals represent latent factors that are comprised of shared genetic variance between certain sets of measured indicators (i.e. measured risk factors). The genetic variance of each risk factor indicator is depicted as a circle, and the residual genetic variance for the indicator that is not captured by the latent factor is depicted as a small oval with the corresponding standardized variances and standard errors. The unidirectional arrows between the latent factors and indicators represent standardized loadings with the corresponding standard errors in parentheses. The double-headed arrows between latent factors represent inter-factor correlation coefficients with standard errors. ALC, alcohol intake frequency (*n* = 420 008); LED, less education (*n* = 416 316); SI, social isolation (*n* = 419 219); SES, socioeconomic status (*n* = 420 035); MDD, major depressive disorder (*n* = 173 005); LON, loneliness (*n* = 413 936); INS, insomnia (*n* = 386 988); T2DM, Type 2 diabetes mellitus (*n* = 936 700); BFP, body fat percentage (*n* = 412 960); MAP, mean arterial pressure (*n* = 417 001); EYE, diagnosis of eye disorder/problem (*n* = 135 363); SMK, smoking (*n* = 632 802).

Since previous studies demonstrate that there is substantial genetic intercorrelation between many complex traits, we also tested whether there are subclusters of genetic pathways shared between the risk factors that display differential associations with Alzheimer’s disease and schizophrenia compared with when risk factors are analysed separately. Using multivariable LD score regression, we found that the included risk factors were highly genetically correlated with one another (Supplementary Table 7) and an EFA in the odd autosomes found that four correlated latent factors best defined this underlying genetic correlation, capturing 55.4% of the total genetic variance between the 13 risk factors (Supplementary Table 8). We used CFA to assess the fit of a four-factor model where any standardized loadings ≥0.30 from the EFA were specified, which was found to fit the data moderately well [$\chi^{2}$ = 323.31, Akaike information criterion (AIC) = 387.31, comparative fit index (CFI) = 0.90, standardized root mean squared error (SRMR) = 0.05] and continued to fit the data well when we fit the model using all autosomes ($\chi^{2}$ = 646.89, AIC = 710.89, CFI = 0.91, SRMR = 0.05) (Fig. 3 and Supplementary Tables 9–10). However, LDL cholesterol was not included in the CFA model as it did not produce any factor loadings above our pre-defined cut-off for inclusion, indicating that this risk factor is more genetically distinct from other risk factor traits.

**Figure 4:**
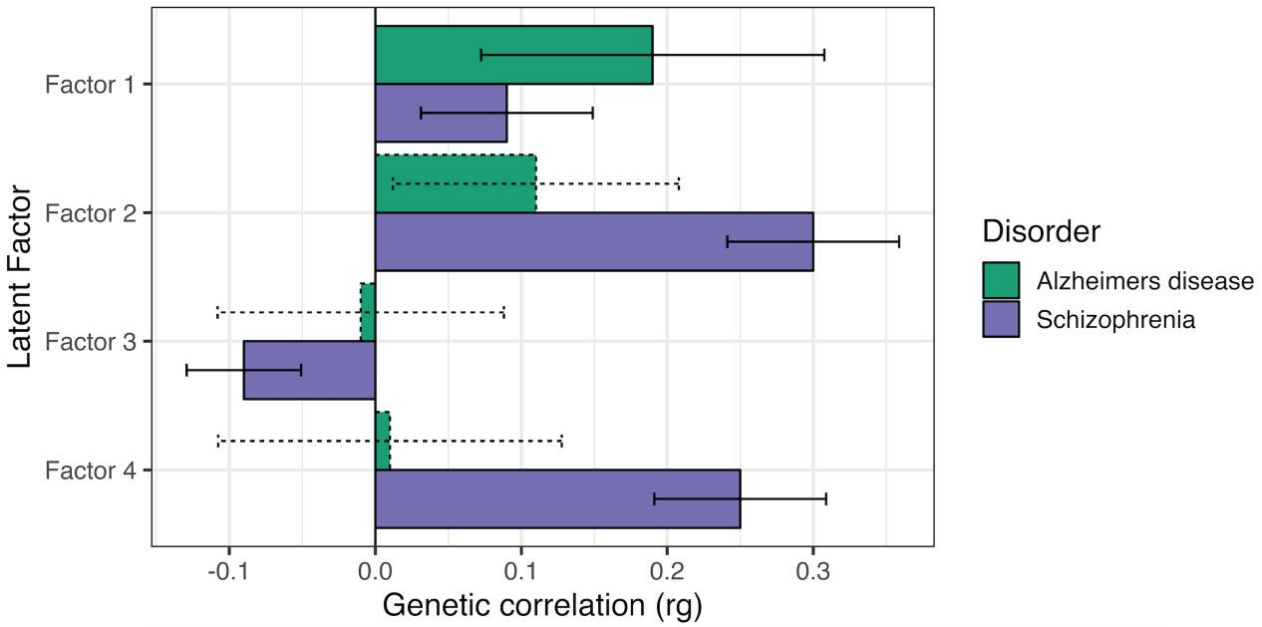
**A comparison of the genetic correlations between Alzheimer’s disease and schizophrenia and the four latent factors of shared genetics between risk factors estimated using multivariable LD score regression.** Solid bars represent significant genetic correlations passing the FDR-corrected *P*-value threshold (*P* < 0.05), whereas dashed lines represent non-significant genetic correlations. Error bars represent the 95% confidence intervals. Alzheimer’s disease, *n =* 54,162; schizophrenia, *n =* 129 325.

We then tested the genetic correlation between each of these latent factors and Alzheimer’s disease and schizophrenia (Fig. 4 and Supplementary Table 11). We found that Factor 1 (shared genetics between high alcohol intake, less education, low socioeconomic status and social isolation) was significantly correlated with both Alzheimer’s disease ($r_{g}$ = 0.19, SE = 0.06, *P*_FDR_ = 1.89 × 10^−03^) and schizophrenia ($r_{g}$ = 0.09, SE = 0.03, *P*_FDR_ = 2.02 × 10^−04^). Factor 2 (shared genetics between major depression, insomnia and loneliness) was significantly correlated with schizophrenia ($r_{g}$ = 0.29, SE = 0.03, *P*_FDR_ = 3.12 × 10^−30^), but not with Alzheimer’s disease ($r_{g}$ = 0.11, SE = 0.05, *P*_FDR_ = 7.12 × 10^−02^). Factor 3 (shared genetics between mean arterial pressure, type 2 diabetes, body fat percentage and diagnosis of eye disorder/problem) was significantly negatively correlated with schizophrenia ($r_{g}$ = −0.09, SE = 0.02, *P*_FDR_ = 3.56 × 10^−06^) and Alzheimer’s disease, although the correlation with the latter was non-significant ($r_{g}$ = −0.01, SE = 0.05, *P*_FDR_ = 8.36 × 10^−01^). Factor 4 (shared genetics between low socioeconomic status, smoking and alcohol intake) was significantly associated with schizophrenia ($r_{g}$ = 0.25, SE = 0.03, *P*_FDR_ = 8.86 × 10^−18^), but not with Alzheimer’s disease ($r_{g}$ = 0.01, SE = 0.06, *P*_FDR_ = 8.36 × 10^−01^). Despite the significant and directionally concordant correlations for both Alzheimer’s disease and schizophrenia with Factor 1, the constrained model $\chi^{2}$ difference test indicated that the magnitude of genetic overlap was significantly different for Alzheimer’s disease and schizophrenia (model $\chi^{2}\;$ difference *P*_FDR_ = 1.89 × 10^−20^) (Supplementary Table 11).

## Discussion

Our findings provide novel evidence that the association seen epidemiologically between schizophrenia and Alzheimer’s disease may, at least in part, be driven by pleiotropic genetic pathways with shared risk factors rather than via direct pathways between the two disorders. Whilst Alzheimer’s disease and schizophrenia were each genetically correlated with several risk factors, the only risk factor that was significantly correlated with both disorders was loneliness. Colocalization analysis of the loneliness-related genomic risk loci indicated that the genetic association between loneliness and Alzheimer’s disease may be predominantly driven by a shared causal variant within the rs12364432 risk locus, whereas schizophrenia is likely to be associated with an independently causal variant in this region and more wide-ranging polygenic effects spread across the genome. In addition, our genomic factor analysis results showed that the genetic variance shared between high alcohol intake, low educational attainment, socioeconomic deprivation and social isolation is significantly associated with both schizophrenia and Alzheimer’s disease.

A key finding of this study is that loneliness displays strong evidence for having a genetic association with both schizophrenia and Alzheimer’s disease. The polygenic overlap between loneliness and schizophrenia has been established in previous papers.^34,35^ An observational study found that although the influence of loneliness on other health traits is similar between people with and without schizophrenia, loneliness is much more prevalent in patients with schizophrenia compared with the rest of the population.^36^ This increased prevalence could therefore lead to increased prevalence of other outcomes that are associated with loneliness (such as dementia) in patients with schizophrenia, even when the two disorders are not directly causally linked.

In contrast, whilst observational evidence shows a positive association between Alzheimer’s disease and loneliness,^37-40^ evidence for their genetic association is more controversial. For example, a UK Biobank data study found a genetic association between dementia and loneliness; however, after adjusting for covariates such as depressive symptoms, education and social deprivation, the results were no longer significant.^41^ Another study found that the cumulative impact of being both socially isolated and a genetic predisposition for loneliness could predispose individuals to dementia by affecting brain health and accelerating brain ageing processes.^42^ Loneliness has been implicated in several systemic and brain pathways that affect our response to stressors. This means that lonely individuals are more vulnerable to processes that affect cognitive decline including increased neuroinflammation, brain atrophy and impaired neuroplasticity.^43^ Despite this, whether loneliness is a contributor to the pathogenesis of Alzheimer’s disease or simply a prodromal symptom of impaired brain processes remains unclear.^40^

Our findings suggest that the genetic correlation between loneliness and Alzheimer’s disease may be driven by a shared causal variant within a loneliness risk locus on Chromosome 11 (lead SNP = rs12364432; effect allele = A; non-effect allele G). Interestingly, this locus was positionally mapped to 16 genes by FUMA, which included *SPI1*, a gene that has consistently been implicated as a risk locus in Alzheimer’s disease GWAS^12,20,21^ and was recently identified as a risk locus for the cognitive aspects of frailty.^44^ Furthermore, the same rs12364432 risk allele has previously been shown to be associated with the loneliness measure in a GWAS of neuroticism.^45^ In contrast, a GWAS-by-subtraction study identified the non-effect allele of this variant as being associated with cognitive aspects of educational attainment.^46^ Together, these findings suggest that this region may be a promising region for more detailed follow-up studies to better understand the underlying biology of the association seen between loneliness and Alzheimer’s disease.

Genomic factor analysis identified four moderately correlated latent factors of shared genetics between the 13 included risk factors. Factor 1 was composed of the genetic overlap between socioeconomic-related risk factors (high alcohol intake, less education, socioeconomic deprivation and social isolation), Factor 2 was composed of shared genetics between mood-related traits (major depression disorder, insomnia and loneliness), Factor 3 was composed by the genetic overlap between cardiometabolic traits and eye disorders (mean arterial pressure, Type 2 diabetes mellitus, body fat percentage and diagnosis of eye disorder/problem) and Factor 4 was composed of the genetic overlap between smoking-related traits (smoking, high alcohol intake and socioeconomic deprivation).

A key finding from our study was that the shared genetic variance between socioeconomic-related risk factors captured by Factor 1 has a significant genetic overlap with both schizophrenia and Alzheimer’s disease, compared with the shared genetics between the other three latent factors. This is in line with previous studies that have demonstrated schizophrenia to be genetically correlated with socioeconomic deprivation^47^ and social isolation.^35^ In contrast, the genetic correlation with Alzheimer’s disease may be largely driven by its association with lower educational attainment given the more established genetic link between these two traits compared to the other risk factors.^48^ However, given that the risk factors that make up Factor 1 all display high loadings that are comparable to the loading for educational attainment, we can conclude that there are shared pathways between education and these other risk factors that may link to both schizophrenia and Alzheimer’s disease through pleiotropic pathways. Future research to define the underlying biology of these shared pathways would help to ascertain whether the genetic covariance between these risk factors create a brain environment that is more susceptible to the neuropathological processes that underpin Alzheimer’s disease and schizophrenia.

Interestingly, Alzheimer’s disease did not significantly correlate with Factor 2, despite having a significant correlation with loneliness. The genetics of Factor 2 were largely composed of major depressive disorder genetics, which shares a strong component with loneliness and, to a lesser extent, insomnia. This may explain the higher genetic correlation point estimate for schizophrenia, as the shared genetic liability between schizophrenia and depression has been extensively demonstrated.^14,49^ Therefore, it is possible that whilst schizophrenia has a strong genetic correlation with psychiatric traits, the genetic pathways that link schizophrenia and loneliness are more relevant for the risk of developing Alzheimer’s disease.

Factor 3 was negatively correlated with schizophrenia and Alzheimer’s disease, although the correlation with the latter was non-significant. Body mass index has been negatively associated with schizophrenia in previous studies.^15,50^ Although obesity is commonly seen in patients with schizophrenia due to the high prevalence of metabolic syndrome,^7^ this negative loading suggests that metabolic syndrome in these patients may depict a predominantly environmental association, potentially influenced by antipsychotic medication, an unhealthy lifestyle and chronic stress.^50^ Whilst poor visual impairment has been associated with an increased risk of psychosis, a recent Mendelian randomization (MR) study found that schizophrenia was a causal risk factor for visual impairment, but not the other way around.^51^ Given that the commonest eye disorders—glaucoma, cataracts, diabetic retinopathy and age-related macular degeneration—are associated with poor metabolic health and metabolic risk factors, it is possible that people with schizophrenia are more likely to develop eye disorders due to their higher risk of metabolic syndrome.^51,52^

Furthermore, whilst the epidemiological association between Alzheimer’s disease and metabolic risk factors has been extensively shown,^6,53^ this is not true for genetic studies, where causation has not been clearly demonstrated. For example, MR studies looking at the correlation between Alzheimer’s disease and blood pressure have found discordant results (no association versus a protective association).^54,55^ The protective association found may be due to the inclusion of proxy Alzheimer’s disease cases which introduce survival bias and may give rise to spurious protective effects. In addition, studies looking at the genetic correlation between adiposity and Alzheimer’s disease have not been able to demonstrate a statistically significant association.^56-58^ A more recent study did not show a correlation with body fat percentage.^59^ Furthermore, type 2 diabetes has not been demonstrated to be a direct causal risk factor for Alzheimer’s disease—although its role in other dementia forms, such as vascular dementia, is expected to be stronger. Similarly, visual impairment, especially cataracts, has been shown to increase the risk of vascular dementia by 2-fold in an MR study.^60^ Our findings are therefore in line with other studies and also show that there is no evidence that metabolic traits drive the genetic association between schizophrenia and Alzheimer’s disease.

Finally, Factor 4 was significantly correlated with schizophrenia but not with Alzheimer’s disease. A recent MR study showed that lower household income was associated with a higher risk of developing schizophrenia and depression.^61^ In addition, SNPs associated with the Townsend deprivation index (marker for socioeconomic status) have been shown to also be associated with schizophrenia.^47^ Schizophrenia has also been demonstrated to be genetically correlated with different smoking phenotypes from nicotine dependence, to cigarettes per day^62^ and shared genetic loci have been identified.^63^ In contrast, the epidemiological association between Alzheimer’s disease and smoking is controversial, with some studies showing no association between the two,^64^ despite it being highlighted as one of the 14 modifiable Alzheimer’s disease risk factors by the Lancet Commission.^6^ This may be, in part, due to survival bias, but genetic studies have also not been able to demonstrate an association.^55,65^ However, the genetic association between Alzheimer’s disease and deprivation has been shown in a recent study.^66^ Whilst the association of Alzheimer’s disease with Factor 4 was not significant, socioeconomic-related risk factors likely play a role in the genetics of Alzheimer’s disease, as seen by the positive loading onto Factor 1, and the non-significant loading may be explained by its association with smoking, which is less clear.

### Limitations

This study should be interpreted in the light of several limitations. First, whilst higher rates of dementia are observed in patients with schizophrenia,^1,2,67^ Alzheimer’s disease was the only dementia type considered in this study. The inclusion of other types of dementia, especially frontotemporal dementia,^68,69^ and Alzheimer’s disease with psychosis^4,70^ would have further helped to disentangle the association between schizophrenia and dementia. Schizophrenia has been linked with the behavioural and familial forms of frontotemporal dementia, and specific genes, such as progranulin, have been associated with the two disorders.^71-73^ Furthermore, it is possible that the dementia observed in patients with schizophrenia does not fit the current dementia nosology, alluding to Emil Kraepelin’s original views of schizophrenia as a *dementia praecox.*^74^ Post-mortem studies of patients with schizophrenia have shown that only a small percentage of cases meet the neuropathological criteria for Alzheimer’s disease^75,76^ and that amyloid-beta pathology is not associated with cognitive impairment in schizophrenia.^77^ Hence, dementia in schizophrenia may be its own subtype, possibly mediated by genetic factors,^78,79^ by the high rates of cardiometabolic-,^7^ socioeconomic-^29,80^ and lifestyle-related risk factors^9,28,81-83^ observed in patients with schizophrenia, by the disease’s own pathological processes^74^ or by a combination of these factors. Nevertheless, the lack of well-powered, publicly available GWAS summary statistics for other dementia subtypes meant we were unable to include them in this study.

Second, this study only included samples of European ancestry due to the lack of publicly available GWAS summary statistics based on non-European populations for all the included phenotypes in this study. This limits the generalizability of our findings. Additionally, since the prevalence of the included risk factors is likely to vary between non-European ancestries, owing to differences in cultural practices, it will be necessary to conduct similar work within multiple populations globally that carefully considers which mediating risk factors are most relevant to that particular population.

Third, it is possible that our correlation estimates between schizophrenia and the measured risk factors with Alzheimer’s disease might be impacted by survival bias, which can produce seemingly protective effects in genetic analyses that include Alzheimer’s disease as the outcome.^84^ This issue has been shown to be especially problematic when the more recent GWAX samples are used.^84,85^ A recent Mendelian randomization study by Liu *et al*.^86^ showed that there is a significant genetic heterogeneity when proxy cases are used, thereby reducing the statistical power of GWAS with proxies. One reason for this might be that proxy cases are more likely to include general dementia diagnoses which can introduce noise to the genetic pool.^22^ With these known caveats in mind, we therefore chose to use the smaller, but more reliably classified case–control Alzheimer’s disease samples for the current study.

## Conclusion

This was the first study to model the shared genetic architecture between Alzheimer’s disease, schizophrenia and associated risk factors using Genomic SEM. We found that loneliness is genetically correlated with both schizophrenia and Alzheimer’s disease, but whilst schizophrenia displays broader polygenic genetic correlation with loneliness, the association between loneliness and Alzheimer’s disease may be driven by a shared causal variant in the locus on Chromosome 11. We also found that the shared genetic pathways between alcohol intake, educational attainment, socioeconomic deprivation and social isolation may share a common component with both schizophrenia and Alzheimer’s disease pathways compared with trait-specific mechanisms. This study highlights the need for more research in the field of dementia in schizophrenia and the importance of understanding the genetics behind observational associations.

## Supplementary Material

fcaf112_Supplementary_Data

## Data Availability

The GWAS summary statistics that we used are available at the following locations: https://pgc.unc.edu/for-researchers/download-results/(schizophrenia and major depressive disorder). https://dss.niagads.org/datasets/ng00036/(Alzheimer’s disease—Lambert *et al.* 2013). Temporarily unavailable as the dataset is being transitioned to the NIAGADS Data Sharing Service. https://www.ebi.ac.uk/gwas/publications/30820047 (Alzheimer’s disease—Kunkle *et al.*^19^). https://pan.ukbb.broadinstitute.org (mean arterial pressure, body fat percentage, alcohol intake, less education, socioeconomic deprivation, loneliness, social isolation, LDL cholesterol and diagnosis of an eye disorder/problem). http://diagram-consortium.org/downloads.html (type 2 diabetes mellitus). https://genome.psych.umn.edu/index.php/GSCAN (smoking). https://ctg.cncr.nl/software/summary_statistics (insomnia). The Hapmap3 SNPs and the LD weights and scores are available to download from: https://utexas.app.box.com/s/vkd36n197m8klbaio3yzoxsee6sxo11v. We used the STREGA checklist when writing our report.^87^ The code scripts for this study will be made available online (https://github.com/IsyFoote) upon publication
