# Supplementary material for: Examining the genetic relationship between Alzheimer’s disease, schizophrenia and their shared risk factors using genomic structural equation modelling
Source: Brain Commun. 2025 Apr 10;7(2):fcaf112. doi: 10.1093/braincomms/fcaf112 (PMC11981896; doi:10.1093/braincomms/fcaf112)
Supplement: fcaf112_Supplementary_Data [file fcaf112_supplementary_data.zip › Supplementary_Materials.pdf]

Supplementary Materials

Supplementary Methods

Trait Selection

In this section we briefly review our rationale for including the risk factors we used in our analyses.

Body fat percentage, mean arterial pressure, LDL cholesterol and type 2 diabetes mellitus

Metabolic syndrome, a combination of central obesity, hypertension, low high-density lipoprotein cholesterol, elevated triglycerides, and hyperglycaemia, is very common in patients with schizophrenia.[1] This may occur as a side-effect of anti-psychotic medication or poor general health.[1] The association between body mass index (BMI) and schizophrenia occurs in both directions. While central obesity is a common feature of metabolic syndrome, low BMI in early life as well as low-birth weight have been associated with an increased risk of schizophrenia.[2] Furthermore, midlife hypertension,[3] midlife dyslipidaemia[4] and obesity (BMI $\geq$ 30),[5] and the duration and severity of type 2 diabetes are associated with an increased risk of dementia.[3]

In this paper, body fat percentage was used instead of BMI because BMI can be highly influenced by age, race, height, and sex and therefore, can be easily overestimated or underestimated.[6] Body fat percentage is thought to be more closely related to obesity.[6] Similarly mean arterial pressure was used instead of systolic and diastolic blood pressure, because it is a measure of central blood pressure, rather than peripheral, and encompasses both the diastolic and the systolic components of blood pressure. Mean arterial pressure has been shown to be genetically influenced and a predictor of hypertension.[7]

High alcohol intake

Excessive alcohol consumption has been associated with an increased risk of dementia.[3] Alcohol leads to structural brain changes, which can result in cognitive and executive dysfunction.[3] Similarly, comorbid substance use is very common in schizophrenia, including alcohol use disorders.[8] Alcohol-induced psychosis can potentiate the transition to schizophrenia.[9]

Insomnia

Insomnia is the commonest sleep disturbance[10] and has been associated with Alzheimer's disease and schizophrenia. Insomnia in both mid and late life may increase the risk of dementia.[11] In addition, patients with schizophrenia often have disturbed sleep, lower sleep efficacy and shorter sleep duration.[12]

Smoking initiation (smoking)

Despite the difficulties in determining the association between dementia and smoking, due to survival bias and competing risk, smoking has been linked to all-cause dementia.[13,14] Moreover, studies have demonstrated that smokers and those exposed to prenatal tobacco are more likely to be diagnosed with schizophrenia.[15]

Loneliness/isolation and low social/leisure activity

Increased social contact is considered a protective factor for dementia, as having an active social life may contribute to cognitive reserve and therefore improve cognitive outcomes.[16] Schizophrenia is associated with childhood social withdrawal[17] and, later in life, patients often have smaller social networks consisting of mostly family members and carers.[18]

Loneliness was considered as a separate trait because it reflects the subjective component of social isolation – socially isolated individuals are not necessarily lonely. Loneliness is positively associated with psychotic disorders, including schizophrenia[19] and it is also associated with dementia and all-cause mortality.[20] One explanation for how loneliness may increase the risk of schizophrenia is that lonely individuals are more likely to find the world around them more threatening and have more negative social interactions which may contribute to an increased risk of paranoia, and the development of psychotic illnesses.[21]

Less education

Less educational attainment is associated with an increased risk of dementia.[22] Likewise, individuals with schizophrenic spectrum disorders have poorer academic achievements by age sixteen.[23] Exposure to high education and high polygenic risk scores for educational attainment are linked with a reduced risk of Alzheimer's disease and schizophrenia, respectively.[22,24]

Socioeconomic deprivation

This trait was measured through the Townsend deprivation index, which considers unemployment, overcrowded households and not owning a car or a house. It is a widely used deprivation score in UK-based populations.[25] Therefore, this was chosen as a measure of low socioeconomic status for our study. While socioeconomic status has traditionally been considered an environmental trait, a genotype-environmental correlation has been proposed. This theory suggests that individuals respond differently to environmental factors based on their genotype.[25,26] Deprivation is associated with both higher rates of dementia[27] and increased mortality.[28] Likewise, individuals who develop schizophrenia are more likely to have been born in deprived areas.[29]

Major depressive disorder

Depressed individuals are more likely to be diagnosed with Alzheimer's disease.[30] However, this relationship is complex because depression is also part of the dementia prodrome.[30,31] In contrast, schizophrenia and major depressive disorder are strongly genetically correlated[32] and the prevalence of depression in patients with schizophrenia has been estimated to be around 40%.[33]

Diagnosis of eye disorder/problem

Untreated vision loss has been highlighted as one of the modifiable risk factors for dementia,[34] specifically visual impairment, cataracts and diabetic retinopathy have been associated with an increased risk of developing all-cause dementia.[35] Visual impairment (of any degree) is associated with the development of schizophrenia and psychotic symptoms.[36,37]

Sample characteristics of the included traits

In this section we describe the sample characteristics of the genome-wide association study (GWAS) summary statistics that we used in this study. Please note that continuous traits do not require population prevalence estimates in Genomic Structural Equation Modelling, so these are not mentioned when a trait is continuous. Furthermore, in cases where a case-control trait was a meta-analysed sample, we calculated the effective sample size to account for ascertainment bias between samples (see main paper Methods), so the sample prevalence is 50%.[38]

Published genome-wide association studies

Alzheimer's disease

We used the GWAS summary statistics from Stage 1 of the International Genomics of Alzheimer's Project (IGAP) by Lambert and colleagues for our main study analysis.[39] A

meta-analysis was conducted with data from four consortia: ADGC (10,273 cases and 10,892 controls), CHARGE (1,315 cases and 12,968 controls), EADI (2,243 cases and 6,017 controls), and GERAD (3,177 cases and 7,277 controls), producing a total of 17,008 cases and 37,154 controls and a total sample size of 54,162.

We also used the Alzheimer's disease GWAS by Kunkle et al.[40] This GWAS used an expanded sample size of the datasets included in the Lambert GWAS, with 17 new datasets and a total sample of 63,926 individuals (21,982 cases and 41,944 controls).

For both Alzheimer's disease GWAS the sample prevalence was 50% and the population prevalence 7.5% was based on the age-specific prevalence of Alzheimer's disease in individuals aged between 70-84 years using data from the 2019 Global Burden of Disease Study.[41] As this study had calculated sex-specific prevalences, we used the average of these estimates for our analysis and used this age range because it coincided with the mean age at assessment of the samples in the Alzheimer's disease GWAS summary statistics.[42]

Schizophrenia

The summary statistics from the most recent schizophrenia GWAS by Trubetskoy et al.[43] were used. This was comprised of 90 cohorts making up a total sample size of 161,405 individuals (67,390 cases of schizophrenia and 94,105 controls). However, as the dataset included a mixture of individuals from different ancestries, we only included the probands of European ancestry making up a total sample size of 129,325 individuals of which 55,193 were cases and 74,132 were controls. The sample prevalence was 50% and the population prevalence was 1%.[44]

Insomnia

The GWAS summary statistics from Watanabe et al., were used with a total sample size of 386,988 individuals (109,548 cases and 277,440 controls).[45] The cohorts included in the original study were from the UK biobank study and 23andMe, however, the 23andMe sample were not included in the present study due to consent requirements. This phenotype was assessed through the question "Do you have trouble falling asleep at night or do you wake up in the middle of the night?". There were four available answers "never/rarely", "sometimes", "usually", and "prefer not to answer", and participants were expected to reply based on the previous 4 weeks. People that answered "usually" were considered cases, and those who answered "prefer not to say" were excluded. The sample prevalence was 28% and the population prevalence 32%.[46]

Major depressive disorder

The GWAS summary statistics from the depression GWAS by Wray and colleagues were used.[47] This was comprised of 7 cohorts: PGC29 (16,823 cases and 25,632 controls), deCODE (1,980 cases and 9,536 controls), GenScotland (997 cases and 6,358 controls), GERA (7,162 cases and 38,307 controls), iPSYCH (18,629 cases and 17,841 controls), UK Biobank (14,260 cases and 15,480 controls), and 23andMe (75,607 cases and 231,747 controls). The summary statistics excluded the 23andMe cohort as this requires participant consent. Therefore, our sample included a total of 59,851 cases and 113,154 controls, for a total sample size of 173,005. Major depressive disorder was measured differently in all cohorts ranging from diagnostic interviews to patient records and self-reported symptoms. The sample prevalence was 50% and the population prevalence 21%.[48]

Smoking initiation

We used the GWAS summary statistics from Liu et al. with a total sample size of 632,802 (223,631 cases and 409,171 controls).[49] This dataset included cohorts from the GSCAN consortium (60 cohorts) and the UK Biobank cohort but excluded the 23andMe data as these data are not publicly available. This is a binary phenotype which included people who reported ever being a smoker (includes current and ex-smokers) and people who reported never being a smoker.

Type 2 diabetes mellitus

The summary statistics from Mahajan et al.[50] were used. Given that this was a multi-ancestry GWAS we only included the summary statistics from European descent individuals who were part of the DIAMANTE consortium. This comprised a total of 82,884 cases and 853,816 controls for a total sample size of 936,700. The sample prevalence was 50% and then population prevalence was 8%.[51]

UK Biobank traits (PanUKB)

All the remaining traits used GWAS summary statistics provided by the Neale lab in their PanUKB data release (European ancestry samples). These include UK Biobank data and are publicly available to download (https://pan.ukbb.broadinstitute.org). The UK biobank study is a large prospective study with over 500,000 participants that collects phenotypic and genotypic information from individuals to better understand the effects of lifestyle, environmental, and genomic factors on the risk of developing common diseases. This study includes individuals aged 49-60, recruited between 2006-2010.[52,53]

Body fat percentage (BFP)

The BFP phenotype (UK Biobank Data Showcase code 23099) was measured by an impedance measurement and was arranged in increments of 0.1%. This phenotype was continuous and was rank normalised. The total sample size was 412,960.

Diagnosis of eye disorder/problem

The diagnosis of an eye disorder (UK Biobank Data Showcase code 6148) was assessed based on the questions “Has a doctor told you that you have any of the following problems with your eyes?”. The answers included “diabetes related eye disease”, “glaucoma”, “injury/trauma resulting in loss of vision”, “cataracts”, “macular degeneration” and “other serious eye conditions”. Those that answered “none of the above” were counted as controls. This was a binary phenotype with a total sample size of 135,363 (30,103 cases and 105,233 controls).

High alcohol intake

The alcohol intake frequency phenotype (UK Biobank Data Showcase code 1558) was assessed based on the question “How often do you drink alcohol?”. The answers included “daily or almost daily”, “three or four times a week”, “once or twice a week”, “one to three times a month”, “special occasions only” and “prefer not to say”. Those who replied “prefer not to say” were not included in the analysis. This phenotype was ordinal and had a total sample size of 420,008.

LDL cholesterol

The LDL-C phenotype was measured through blood samples (UK Biobank Data Showcase code 30780, adjusted using fields 6153 & 6177). This was adjusted by use of cholesterol lowering medication. This was a continuous trait with a total sample size of 398,402.

Less education

The less education phenotype (UK Biobank Data Showcase code 6138) was assessed based on the question “which of the following qualifications do you have?”. The possible answers were “College or university degree”, “A levels/AS levels or equivalent”, “O levels/GCSEs or equivalent”, “CSEs or equivalent”, “NVQ or HNC or HNC or equivalent”, “other professional qualifications e.g., nursing, teaching”, “none of the above”, or “prefer not to say”. Those who answered with “prefer not to say” were excluded from the analysis and those who answered, “none of the above”, were used as proxy for less education.

This was a binary phenotype composed of 73,293 cases and 343,023 controls, accounting for a total sample size of 416,316. The sample prevalence was 18% and the population prevalence 27%.[54]

Loneliness/isolation

This phenotype (UK Biobank Data Showcase code 2020) was assessed based on the question “Do you often feel lonely?”. The possible answers were “Yes”, “No”, “Do not know” or “Prefer not to say”. Those who answered “Prefer not to say” were excluded from the analysis. This was a binary phenotype composed of 74,098 cases and 339,838 controls, accounting for a total sample size of 413,936. The sample prevalence was 18% and the population prevalence was 29%.[55]

Low social/leisure activity

This phenotype (UK Biobank Data Showcase code 6160_100) was measured through the question “Which of the following do you attend once a week or more often? (you can select more than one)”. The possible answers were “sports club or gym”, “pub or social club”, “religious group”, “adult education class”, “other groups activity”, “none of the above” or “prefer not to say”. Those who chose the latter option were excluded from the analysis. This was a binary phenotype comprised of 126,966 cases and 292,253 controls, for a total sample size of 419,219. The sample prevalence was 30% and the population prevalence was 53%.[56]

Mean arterial pressure

This phenotype (UK Biobank Data Showcase codes data fields 4080, 93, 4079 & 94; adjusted using 6153 & 6177) was measured by combining the automated and manual blood pressure readings and adjusting for anti-hypertensive medication. The following equation was used - systolic blood pressure (SBP) + diastolic blood pressure (DBP)/3. The SBP data was obtained from code fields 4080 and 93, and the DBP obtained from code fields 4079 and 94. This was adjusted for the use of anti-hypertensive medication using code fields 6153_2 and 6177_2. This was a continuous trait and the total sample size was 417,001.

Socioeconomic deprivation

The Townsend deprivation index phenotype (UK Biobank Data Showcase code 189) was calculated before participants joined the UK biobank study and was based on the preceding national census output areas. The participants were given a score based on

the output area where their postcode was located. This was a continuous trait that was rank normalised. The total sample size was 420,035.

Supplementary Results

Our sensitivity analysis using the Kunkle et al GWAS data for Alzheimer's disease generally demonstrated a similar pattern of results compared to the main analyses conducted with the Lambert et al GWAS data. However, Alzheimer's disease was only significantly correlated with lower educational attainment (r_g = 0.17; *SE* = 0.04, P_{FDR} = $1.12x10^{-03}$), but not with increased loneliness as seen in the main analysis (**Supplementary Table 12**). The model of the genetic correlation between each latent factor showed that Alzheimer's disease was nominally associated with Factor 1, but this effect did not survive the multiple testing corrected significance threshold (**Supplementary Table 13**). Despite the lack of statistically significant genetic correlations in the Kunkle data, the general pattern of findings was the same as our main analysis.

Bibliography

1. Vancampfort D, Stubbs B, Mitchell AJ, et al. Risk of metabolic syndrome and its components in people with schizophrenia and related psychotic disorders, bipolar disorder and major depressive disorder: a systematic review and meta-analysis. *World Psychiatry*. Oct 2015;14(3):339-47. doi:10.1002/wps.20252
2. Zammit S, Rasmussen F, Farahmand B, et al. Height and body mass index in young adulthood and risk of schizophrenia: a longitudinal study of 1 347 520 Swedish men. *Acta Psychiatr Scand*. Nov 2007;116(5):378-85. doi:10.1111/j.1600-0447.2007.01063.x
3. Livingston G, Huntley J, Sommerlad A, et al. Dementia prevention, intervention, and care: 2020 report of the Lancet Commission. *Lancet*. Aug 8 2020;396(10248):413-446. doi:10.1016/s0140-6736(20)30367-6
4. Wee J, Sukudom S, Bhat S, et al. The relationship between midlife dyslipidemia and lifetime incidence of dementia: A systematic review and meta-analysis of cohort studies. *Alzheimers Dement (Amst)*. Jan-Mar 2023;15(1):e12395. doi:10.1002/dad2.12395
5. Albanese E, Launer LJ, Egger M, et al. Body mass index in midlife and dementia: Systematic review and meta-regression analysis of 589,649 men and women followed in longitudinal studies. *Alzheimers Dement (Amst)*. 2017;8:165-178. doi:10.1016/j.dadm.2017.05.007
6. Escobedo-de la Peña J, Ramírez-Hernández JA, Fernández-Ramos MT, González-Figueroa E, Champagne B. Body Fat Percentage Rather than Body Mass Index Related to the High Occurrence of Type 2 Diabetes. *Arch Med Res*. Aug 2020;51(6):564-571. doi:10.1016/j.arcmed.2020.05.010
7. Wain LV, Verwoert GC, O'Reilly PF, et al. Genome-wide association study identifies six new loci influencing pulse pressure and mean arterial pressure. *Nat Genet*. Sep 11 2011;43(10):1005-11. doi:10.1038/ng.922
8. Hunt GE, Large MM, Cleary M, Lai HMX, Saunders JB. Prevalence of comorbid substance use in schizophrenia spectrum disorders in community and clinical settings, 1990-2017: Systematic review and meta-analysis. *Drug Alcohol Depend*. Oct 1 2018;191:234-258. doi:10.1016/j.drugalcdep.2018.07.011
9. Murrie B, Lappin J, Large M, Sara G. Transition of Substance-Induced, Brief, and Atypical Psychoses to Schizophrenia: A Systematic Review and Meta-analysis. *Schizophr Bull*. Apr 10 2020;46(3):505-516. doi:10.1093/schbul/sbz102
10. Ferrie JE, Kumari M, Salo P, Singh-Manoux A, Kivimäki M. Sleep epidemiology--a rapidly growing field. *Int J Epidemiol*. 2011:1431-7. vol. 6.
11. Sindi S, Kåreholt I, Johansson L, et al. Sleep disturbances and dementia risk: A multicenter study. *Alzheimers Dement*. Oct 2018;14(10):1235-1242. doi:10.1016/j.jalz.2018.05.012
12. Chan MS, Chung KF, Yung KP, Yeung WF. Sleep in schizophrenia: A systematic review and meta-analysis of polysomnographic findings in case-control studies. *Sleep Med Rev*. Apr 2017;32:69-84. doi:10.1016/j.smrv.2016.03.001
13. Chang CC, Zhao Y, Lee CW, Ganguli M. Smoking, death, and Alzheimer disease: a case of competing risks. *Alzheimer Dis Assoc Disord*. Oct-Dec 2012;26(4):300-6. doi:10.1097/WAD.0b013e3182420b6e
14. Zhong G, Wang Y, Zhang Y, Guo JJ, Zhao Y. Smoking is associated with an increased risk of dementia: a meta-analysis of prospective cohort studies with investigation of potential effect modifiers. *PLoS One*. 2015;10(3):e0118333. doi:10.1371/journal.pone.0118333
15. Hunter A, Murray R, Asher L, Leonardi-Bee J. The Effects of Tobacco Smoking, and Prenatal Tobacco Smoke Exposure, on Risk of Schizophrenia: A Systematic Review and Meta-Analysis. *Nicotine Tob Res*. Jan 27 2020;22(1):3-10. doi:10.1093/ntr/nty160

16. Evans IEM, Martyr A, Collins R, Brayne C, Clare L. Social Isolation and Cognitive Function in Later Life: A Systematic Review and Meta-Analysis. *J Alzheimers Dis*. 2019;70(s1):S119-s144. doi:10.3233/jad-180501
17. Matheson SL, Vijayan H, Dickson H, Shepherd AM, Carr VJ, Laurens KR. Systematic meta-analysis of childhood social withdrawal in schizophrenia, and comparison with data from at-risk children aged 9-14 years. *J Psychiatr Res*. Aug 2013;47(8):1061-8. doi:10.1016/j.jpsychires.2013.03.013
18. Seeman MV. Schizophrenia Mortality: Barriers to Progress. *Psychiatr Q*. Sep 2019;90(3):553-563. doi:10.1007/s11126-019-09645-0
19. Michalska da Rocha B, Rhodes S, Vasilopoulou E, Hutton P. Loneliness in Psychosis: A Meta-analytical Review. *Schizophr Bull*. Jan 13 2018;44(1):114-125. doi:10.1093/schbul/sbx036
20. Sutin AR, Stephan Y, Luchetti M, Terracciano A. Loneliness and Risk of Dementia. *J Gerontol B Psychol Sci Soc Sci*. Aug 13 2020;75(7):1414-1422. doi:10.1093/geronb/gby112
21. Rødevand L, Bahrami S, Frei O, et al. Polygenic overlap and shared genetic loci between loneliness, severe mental disorders, and cardiovascular disease risk factors suggest shared molecular mechanisms. *Transl Psychiatry*. Jan 5 2021;11(1):3. doi:10.1038/s41398-020-01142-4
22. Xu W, Tan L, Wang HF, et al. Education and Risk of Dementia: Dose-Response Meta-Analysis of Prospective Cohort Studies. *Mol Neurobiol*. Jul 2016;53(5):3113-3123. doi:10.1007/s12035-015-9211-5
23. Dickson H, Hedges EP, Ma SY, et al. Academic achievement and schizophrenia: a systematic meta-analysis. *Psychol Med*. Sep 2020;50(12):1949-1965. doi:10.1017/s0033291720002354
24. Sørensen HJ, Debost JC, Agerbo E, et al. Polygenic Risk Scores, School Achievement, and Risk for Schizophrenia: A Danish Population-Based Study. *Biol Psychiatry*. Nov 1 2018;84(9):684-691. doi:10.1016/j.biopsych.2018.04.012
25. Ye J, Wen Y, Sun X, et al. Socioeconomic Deprivation Index Is Associated With Psychiatric Disorders: An Observational and Genome-wide Gene-by-Environment Interaction Analysis in the UK Biobank Cohort. *Biol Psychiatry*. Nov 26 2020;doi:10.1016/j.biopsych.2020.11.019
26. Trzaskowski M, Harlaar N, Arden R, et al. Genetic influence on family socioeconomic status and children's intelligence. *Intelligence*. Jan 2014;42(100):83-88. doi:10.1016/j.intell.2013.11.002
27. Cadar D, Lassale C, Davies H, Llewellyn DJ, Batty GD, Steptoe A. Individual and Area-Based Socioeconomic Factors Associated With Dementia Incidence in England: Evidence From a 12-Year Follow-up in the English Longitudinal Study of Ageing. *JAMA psychiatry*. 2018;75(7):723-732. doi:10.1001/jamapsychiatry.2018.1012
28. Korhonen K, Einiö E, Leinonen T, Tarkiainen L, Martikainen P. Midlife socioeconomic position and old-age dementia mortality: a large prospective register-based study from Finland. *BMJ Open*. Jan 6 2020;10(1):e033234. doi:10.1136/bmjopen-2019-033234
29. O'Donoghue B, Roche E, Lane A. Neighbourhood level social deprivation and the risk of psychotic disorders: a systematic review. *Soc Psychiatry Psychiatr Epidemiol*. Jul 2016;51(7):941-50. doi:10.1007/s00127-016-1233-4
30. Ownby RL, Crocco E, Acevedo A, John V, Loewenstein D. Depression and risk for Alzheimer disease: systematic review, meta-analysis, and metaregression analysis. *Arch Gen Psychiatry*. May 2006;63(5):530-8. doi:10.1001/archpsyc.63.5.530
31. Singh-Manoux A, Dugravot A, Fournier A, et al. Trajectories of Depressive Symptoms Before Diagnosis of Dementia: A 28-Year Follow-up Study. *JAMA Psychiatry*. Jul 1 2017;74(7):712-718. doi:10.1001/jamapsychiatry.2017.0660
32. Anttila V, Bulik-Sullivan B, Finucane HK, et al. Analysis of shared heritability in common disorders of the brain. *Science*. Jun 22 2018;360(6395)doi:10.1126/science.aap8757

33. Upthegrove R, Marwaha S, Birchwood M. Depression and Schizophrenia: Cause, Consequence, or Trans-diagnostic Issue? *Schizophr Bull*. Mar 1 2017;43(2):240-244. doi:10.1093/schbul/sbw097
34. Livingston G, Huntley J, Liu KY, et al. Dementia prevention, intervention, and care: 2024 report of the Lancet standing Commission. *Lancet*. Aug 10 2024;404(10452):572-628. doi:10.1016/s0140-6736(24)01296-0
35. Kuźma E, Littlejohns TJ, Khawaja AP, Llewellyn DJ, Ukoumunne OC, Thiem U. Visual Impairment, Eye Diseases, and Dementia Risk: A Systematic Review and Meta-Analysis. *J Alzheimers Dis*. 2021;83(3):1073-1087. doi:10.3233/jad-210250
36. Shoham N, Lewis G, Hayes JF, Silverstein SM, Cooper C. Association between visual impairment and psychosis: A longitudinal study and nested case-control study of adults. *Schizophr Res*. Apr 2023;254:81-89. doi:10.1016/j.schres.2023.02.017
37. Shoham N, Hayes JF, Cooper C, Theodorsson M, Lewis G. Association Between Childhood Visual Acuity and Late Adolescent Psychotic Experiences: A Prospective Birth Cohort Study. *Schizophr Bull*. Mar 1 2022;48(2):325-334. doi:10.1093/schbul/sbab121
38. Grotzinger AD, Fuente J, Privé F, Nivard MG, Tucker-Drob EM. Pervasive Downward Bias in Estimates of Liability-Scale Heritability in Genome-wide Association Study Meta-analysis: A Simple Solution. *Biol Psychiatry*. Jan 1 2023;93(1):29-36. doi:10.1016/j.biopsych.2022.05.029
39. Lambert J-C, Ibrahim-Verbaas CA, Harold D, et al. Meta-analysis of 74,046 individuals identifies 11 new susceptibility loci for Alzheimer's disease. *Nature Genetics*. 2013/12/01 2013;45(12):1452-1458. doi:10.1038/ng.2802
40. Kunkle BW, Grenier-Boley B, Sims R, et al. Genetic meta-analysis of diagnosed Alzheimer's disease identifies new risk loci and implicates Aβ, tau, immunity and lipid processing. *Nat Genet*. Mar 2019;51(3):414-430. doi:10.1038/s41588-019-0358-2
41. Estimation of the global prevalence of dementia in 2019 and forecasted prevalence in 2050: an analysis for the Global Burden of Disease Study 2019. *Lancet Public Health*. Feb 2022;7(2):e105-e125. doi:10.1016/s2468-2667(21)00249-8
42. Escott-Price V, Hardy J. Genome-wide association studies for Alzheimer's disease: bigger is not always better. *Brain Commun*. 2022;4(3):fcac125. doi:10.1093/braincomms/fcac125
43. Trubetskoy V, Pardiñas AF, Qi T, et al. Mapping genomic loci implicates genes and synaptic biology in schizophrenia. *Nature*. Apr 2022;604(7906):502-508. doi:10.1038/s41586-022-04434-5
44. Owen MJ, Sawa A, Mortensen PB. Schizophrenia. *Lancet*. Jul 2 2016;388(10039):86-97. doi:10.1016/s0140-6736(15)01121-6
45. Watanabe K, Jansen PR, Savage JE, et al. Genome-wide meta-analysis of insomnia prioritizes genes associated with metabolic and psychiatric pathways. *Nat Genet*. Aug 2022;54(8):1125-1132. doi:10.1038/s41588-022-01124-w
46. Kerkhof GA. Epidemiology of sleep and sleep disorders in The Netherlands. *Sleep Med*. Feb 2017;30:229-239. doi:10.1016/j.sleep.2016.09.015
47. Wray NR, Ripke S, Mattheisen M, et al. Genome-wide association analyses identify 44 risk variants and refine the genetic architecture of major depression. *Nat Genet*. May 2018;50(5):668-681. doi:10.1038/s41588-018-0090-3
48. Grotzinger AD, Mallard TT, Akingbuwa WA, et al. Genetic architecture of 11 major psychiatric disorders at biobehavioral, functional genomic and molecular genetic levels of analysis. *Nat Genet*. May 2022;54(5):548-559. doi:10.1038/s41588-022-01057-4
49. Liu M, Jiang Y, Wedow R, et al. Association studies of up to 1.2 million individuals yield new insights into the genetic etiology of tobacco and alcohol use. *Nat Genet*. Feb 2019;51(2):237-244. doi:10.1038/s41588-018-0307-5
50. Mahajan A, Spracklen CN, Zhang W, et al. Multi-ancestry genetic study of type 2 diabetes highlights the power of diverse populations for discovery and translation. *Nat Genet*. May 2022;54(5):560-572. doi:10.1038/s41588-022-01058-3

51. Khan MAB, Hashim MJ, King JK, Govender RD, Mustafa H, Al Kaabi J. Epidemiology of Type 2 Diabetes - Global Burden of Disease and Forecasted Trends. *J Epidemiol Glob Health*. Mar 2020;10(1):107-111. doi:10.2991/jegh.k.191028.001
52. Sudlow C, Gallacher J, Allen N, et al. UK biobank: an open access resource for identifying the causes of a wide range of complex diseases of middle and old age. *PLoS medicine*. 2015;12(3):e1001779-e1001779. doi:10.1371/journal.pmed.1001779
53. UK Biobank. Accessed 13/03/2023, 2023. https://www.ukbiobank.ac.uk/
54. Crimmins EM, Saito Y, Kim JK, Zhang YS, Sasson I, Hayward MD. Educational Differences in the Prevalence of Dementia and Life Expectancy with Dementia: Changes from 2000 to 2010. *J Gerontol B Psychol Sci Soc Sci*. Apr 16 2018;73(suppl_1):S20-s28. doi:10.1093/geronb/gbx135
55. Chawla K, Kunonga TP, Stow D, Barker R, Craig D, Hanratty B. Prevalence of loneliness amongst older people in high-income countries: A systematic review and meta-analysis. *PLoS One*. 2021;16(7):e0255088. doi:10.1371/journal.pone.0255088
56. Kotwal AA, Cenzer IS, Waite LJ, et al. The epidemiology of social isolation and loneliness among older adults during the last years of life. *J Am Geriatr Soc*. Nov 2021;69(11):3081-3091. doi:10.1111/jgs.17366
